# Longer amplicons provide better sensitivity for electrochemical sensing of viral nucleic acid in water samples using PCB electrodes

**DOI:** 10.1038/s41598-022-12818-w

**Published:** 2022-05-25

**Authors:** Shruti Ahuja, M. Santhosh Kumar, Ruchira Nandeshwar, Kiran Kondabagil, Siddharth Tallur

**Affiliations:** 1 Centre for Research in Nanotechnology and Science (CRNTS), IIT Bombay, Mumbai, 400076 India; 2 Department of Biosciences and Bioengineering (BSBE), IIT Bombay, Mumbai, 400076 India; 3 Department of Electrical Engineering (EE), IIT Bombay, Mumbai, 400076 India

**Keywords:** Biomedical engineering, Characterization and analytical techniques, Sensors and probes

## Abstract

The importance of monitoring environmental samples has gained a lot of prominence since the onset of COVID-19 pandemic, and several surveillance efforts are underway using gold standard, albeit expensive qPCR-based techniques. Electrochemical DNA biosensors could offer a potential cost-effective solution suitable for monitoring of environmental water samples in lower middle income countries. In this work, we demonstrate electrochemical detection of amplicons as long as ${503}\,\hbox {bp}$ obtained from Phi6 bacteriophage (a popular surrogate for SARS-CoV-2) isolated from spiked lake water samples, using ENIG finish PCB electrodes with no surface modification. The electrochemical sensor response is thoroughly characterised for two DNA fragments of different lengths (${117}\,\hbox {bp}$ and ${503}\,\hbox {bp}$), and the impact of salt in PCR master mix on methylene blue (MB)-DNA interactions is studied. Our findings establish that length of the DNA fragment significantly determines electrochemical sensitivity, and the ability to detect long amplicons without gel purification of PCR products demonstrated in this work bodes well for realisation of fully-automated solutions for in situ measurement of viral load in water samples.

## Introduction

Waterborne viral transmission as a public health hazard has been known since the 1940s, with first documented evidence of transmission of polio and hepatitis E via the water route^1^. The World Health Organization (WHO) has classified several waterborne viral pathogens with moderate to high health significance^2^. Conventional methods for virus detection rely on gold standard qPCR-based techniques that are highly sensitive and specific, but require skilled personnel for running the tests with expensive instruments in laboratories. However, in low middle income countries (LMICs) with limited resources, testing of human samples would very likely be prioritised over environmental water sample surveillance. Therefore, there is a need for alternative low-cost approaches for sustainable, real-time surveillance of water and wastewater samples in LMICs, to serve as early warning of emerging disease outbreak and thereby shield them from harsh socioeconomic impact of viral pandemics^3^. Low-cost nucleic acid electrochemical biosensors could offer a promising potential solution to this unmet need^4^. The principle of operation of many such DNA biosensors in that a complementary strand of DNA is immobilised on the surface of the electrode, with hybridisation occurring when the matching sequence is present within the sample. This can then be transduced into a signal through a wide range of electrochemical techniques with the use of a redox mediator, such as potassium ferri/ferro-cyanide. Methylene Blue (MB) is one such redox active molecule, that is reported to intercalate with double stranded DNA (dsDNA) in addition to more non-specific binding to single stranded DNA^5,6^. The intercalating properties of MB to form MB-DNA complex have made it a popular choice as redox mediator in several electrochemical DNA sensor configurations^5–9^. While MB intercalation with DNA is non-specific and the specificity of such electrochemical sensors heavily relies upon purity of primers used for PCR or isothermal amplification, it is well suited for realising an alternate to qPCR or fluorescence isothermal amplification, based on real-time electrochemical measurement of DNA concentration^9^. In one such implementation, Won et al. modified the surface of a gold electrode with 6-mercapto-1-hexanol (MCH) to measure PCR amplicons with MB in real-time using differential pulse voltammetry (DPV)^9^. In other instance, Ramirez et al. used MB with screen printed electrodes for the detection of SARS-CoV-2 in wastewater with RT-LAMP reaction^10^. Platinum electrodes have also been used for an in-situ electrode during microfluidic PCR platform designed to electrochemically detect amplicons during reactions^8^. All these studies require surface modification of the electrode, thereby implying increased production and operating costs due to specialised storage requirements for stability of these functionalised electrodes.

**Figure 1:**
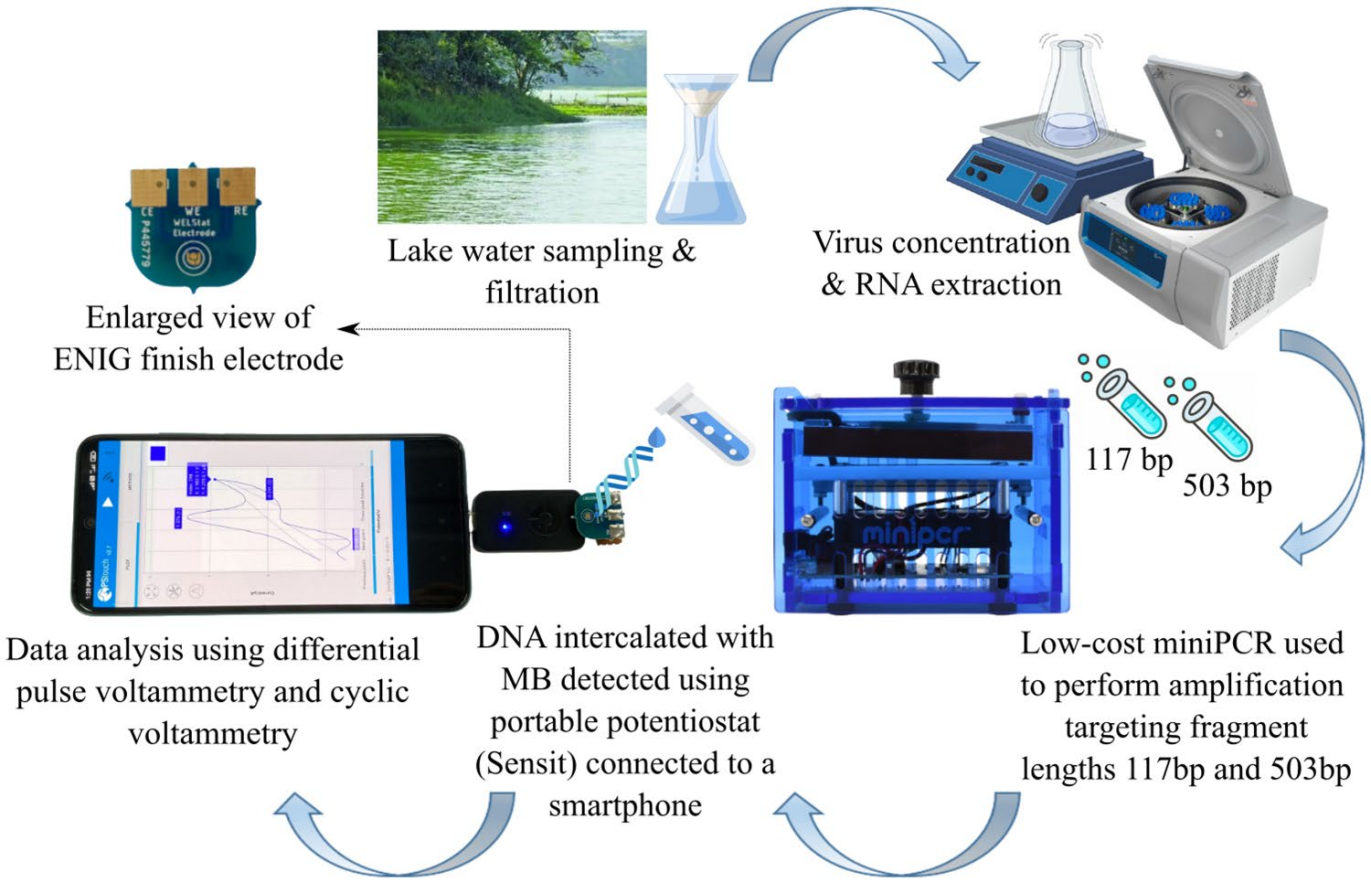
Illustration of work-flow for electrochemical detection of amplicons obtained from virus particles concentrated from lake water samples.

We recently demonstrated electrochemical sensing of SARS-CoV-2 amplicons with low-cost printed circuit board (PCB) electrodes, based on change in DPV and cyclic voltammetry (CV) peak current due to adsorption of MB-DNA complex on the unmodified electrode surface^11^. We reported that longer DNA fragment (N1-N2, ${943}\,\hbox {bp}$) formed using CDC-recommended N1 forward and N2 reverse primers exhibited better linearity in sensor response as compared to the shorter fragment (N1, $72\,\hbox {bp}$) formed using N1 forward and N1 reverse primer set. These studies were reported using DNA dilutions prepared in nuclease free water. The platform was also used for detection of SARS-CoV-2 amplicons in simulated wastewater sample (obtained by spiking total RNA sample with SARS-CoV-2 RNA). The amplification of longer fragments with such heterogeneous samples is difficult, since RNA is susceptible to shearing during isolation and downstream processing^12,13^. Thus, the demonstration of electrochemical sensing of SARS-CoV-2 amplicons in wastewater was limited to the shorter $72\,\hbox {bp}$ N1 fragment^11^.

In this work, we study the feasibility of ENIG PCB based electrochemical sensing of bacteriophage Phi6 concentrated and isolated from lake water samples (illustrated in Fig. 1). Phi6 phage is comparable to SARS-CoV-2 in size (80–100 nm), and also possesses lipid membrane and spike proteins. Because of these reasons, phage Phi6 is a popular surrogate for SARS-CoV-2 and other enveloped pathogenic RNA viruses^14,15^. RNA isolated from the phage particles was used as template for cDNA synthesis, that was then used as template for PCR to obtain two DNA fragments of lengths 117 and 503 base pairs. Given the challenges of amplifying $943\,\hbox {bp}$ N1-N2 fragment in our previous work, we have targeted fragments of intermediate length ($117\,\hbox {bp}$ and $503\,\hbox {bp}$) in this study, based on available primers. The electrochemical sensor response was systematically studied for a wide range of concentration (${10}\,{\hbox {pg}/{\upmu \hbox {l}}}$ to ${20}\,{\hbox {ng}/{\upmu \hbox {l}}}$) for both fragments in presence of MB, and the impact of salt on sensor response was characterised and cross-validated with spectrophotometry measurements. The key contributions of this work are as follows:

- *DNA fragment length and presence of salt in sample strongly influence sensitivity* Our results demonstrate that the electrochemical activity depends on different mechanisms of MB, DNA, and sensor interaction in voltammetric response depending on the DNA concentration and length, with the longer fragment showing higher sensitivity, despite the negative impact of salt on the electrostatic interactions between MB and DNA.
- *DNA concentration determines mechanism of MB-DNA interactions for unmodified electrodes* We demonstrate that different mechanisms of MB-DNA interaction are at play depending on DNA concentration. At DNA concentrations lower than few ${\hbox {ng}/{\upmu \hbox {l}}}$, we observed the electrochemical current response to be dominated by adsorption of MB-DNA on the electrode, whereas at higher DNA concentrations, the electrochemical current response is determined by steric inhibition of redox activity due to intercalation of MB between base pairs of DNA.
- *ENIG PCB based electrochemical sensing of viral nucleic acid from lake water samples* The observations were validated by electrochemical detection of the $503\,\hbox {bp}$ DNA fragment obtained from water sample from Powai lake on IIT Bombay campus spiked with Phi6 phage.
- *Low-cost of implementation, with potential for integration into fully-automated surveillance systems* The technology presented in this work requires no additional gold electroplating atop the ENIG finish PCB, and has long shelf life considering there is no surface modification with alkanethiol layer or immobilisation of oligonucleotides or aptamers on the electrode.

## Materials and methods

### Preparation of target amplicons

Bacteriophage Phi6 is an enveloped dsRNA virus of the *Cystoviridae* family that infects *Pseudomonas syringae*. The genome of Phi6 phage is present in the form of 3 segments: S ($2.95\,\hbox {Kb}$), M ($4.07\,\hbox {Kb}$), and L ($6.37\,\hbox {Kb}$)^16,17^. Since Phi6 phage infects a nonpathogenic BSL-1 strain of Pseudomonas, it is safe to work with and can be cultivated easily in the lab. Bacteriophage Phi6 and its host, *Pseudomonas Syringae*, were procured from Felix d’Herelle Reference Center for Bacterial Viruses, University of Laval, Canada (reference center catalog numbers, HER-102 and HER-1102, respectively). Phi6 phage and its host were revived as per instructions of the Reference Center. Phage Phi6 was purified by plate lysis and elution method^18^ and the final titers obtained had $\approx 10^{12}\,{\hbox {PFU}/\hbox {ml}}$ (Plaque Forming Units/ milliliter). RNA was isolated from the purified phage particles using GenElute™ Universal Total RNA purification kit (Sigma-Aldrich) as per the manufacturer’s instructions. Briefly, ${100}\,{{\upmu \hbox {l}}}$ of purified bacteriophage Phi6 suspension was lysed, and the lysate was loaded on to the spin column to allow the binding of RNA to the resin in the column. RNA was then eluted in ${50}\,{{\upmu \hbox {l}}}$ of elution solution provided with the kit. Concentration of the RNA was estimated by absorbance at $260\,\hbox {nm}$. RNA was stored as aliquots at ${-80}\,{^{\circ }\hbox {C}}$ until further use. ${2}\,{\upmu \hbox {g}}$ of the isolated RNA was used as a template for cDNA synthesis using iScript cDNA synthesis kit (Bio-Rad Laboratories) as per the manufacturer’s instructions. Briefly, the cDNA synthesis reaction included 3 steps: priming at ${25}\,{^{\circ }\hbox {C}}$ for ${5}\,{\hbox {min}}$, reverse transcription at ${46}\,{^{\circ }\hbox {C}}$ for ${20}\,{\hbox {min}}$, and inactivation of reverse transcriptase enzyme at ${95}\,{^{\circ }\hbox {C}}$ for ${1}\,{\hbox {min}}$. The cDNA, when electrophoresed on a 1 % agarose gel, showed three bands corresponding to three RNA segments as expected (data not shown). The following primers were used to amplify two DNA fragments of length 117 and 503 base pairs, using the cDNA as a template for PCR in miniPCR® mini8 thermal cycler:

117-Forward: 5$$^\prime$$-GAATCATATGCGCTACCAAGGCATCAAC-3$$^\prime$$

117-Reverse: 5$$^\prime$$-CATAGAATTCTGGGAGGAGCAGCGGAGA -3$$^\prime$$

503-Forward: 5$$^\prime$$- GAACCATATGACTTTGTACCTGGTCC-3$$^\prime$$

503-Reverse: 5$$^\prime$$- CAACGAATTCTCAGGCGCTTACCTCATC-3$$^\prime$$

The primers for $117\,\hbox {bp}$ and $503\,\hbox {bp}$ corresponded to 1476–1575 nucleotides of segment M, and 458–943 nucleotides of segment L, respectively. All the amplified PCR products were electrophoresed on 1 % agarose gel, and the amplified target DNA was purified using GeneJET gel extraction kit (Thermo Fisher scientific).

### Target amplification from environmental water sample spiked with bacteriophage Phi6

Water from a lake on IIT Bombay campus (Powai lake, Powai, Mumbai) was used for spiking with phage particles. The lake water was filtered through a ${5}\,{\upmu \hbox {m}}$ membrane to remove suspended particles prior to spiking with Phi6 phage. ${1}\,{\hbox {ml}}$ of $10^{6}\,{\hbox {PFU}/\hbox {ml}}$ of Phi6 phage preparation was added to ${100}\,{\hbox {ml}}$ of filtered lake water and gently homogenised for ${10}\,{\hbox {min}}$ at ${4}\,{^{\circ }\hbox {C}}$. A small aliquot of the sample was kept aside for measuring virus load by plaque assay. We tested two different methods for concentrating the spiked Phi6 virus particles: (1) aluminium hydroxide adsorption-precipitation method^19^, that has been validated for concentration of several enveloped RNA viruses from environmental samples, and (2) a polyethylene glycol (PEG) based virus concentration method adapted from Flood et al.^20^. Since recovery efficiency of the PEG-based method was found to be better than the aluminium hydroxide method, PEG based method was used for concentrating the Phi6 particles from the lake water sample.

The PEG method used is as follows: PEG 8000 and $\hbox {NaCl}$ were added to the Phi6-spiked lake water sample to obtain a sample with 8 % PEG 8000 and $0.2\,\hbox {M}$ $\hbox {NaCl}$. The samples were incubated in a shaker at ${4}\,{^{\circ }\hbox {C}}$ for ${4}\,{\hbox {h}}$, followed by centrifugation at $4700\,\hbox {g}$ for ${45}\,{\hbox {min}}$. The supernatant was discarded and the pellet was resuspended in ${1}\,{\hbox {ml}}$ of the same supernatant. All spiking and virus concentration experiments were conducted in triplicate. After concentration, a small aliquot was kept aside for measuring the recovery efficiency by plaque assay method. RNA was isolated as described earlier and eluted in ${40}\,{\upmu \hbox {l}}$ of elution buffer provided with the kit. Since the RNA concentration would differ from sample to sample in the triplicate, ${2}\,{\upmu \hbox {l}}$ of RNA was used for cDNA synthesis for all three samples, irrespective of their concentrations. cDNA synthesis was carried out as described earlier. ${1}\,{\upmu \hbox {l}}$ cDNA was used as a template for ${20}\,{\upmu \hbox {l}}$ PCRs for 35 cycles, to amplify $117\,\hbox {bp}$ and $503\,\hbox {bp}$ fragments. These samples are denoted ‘1:1’ i.e. no dilution. A no-template control (NTC) was set up as a negative control, while positive control (PC) was set up using the cDNA synthesized from RNA isolated from purified phages as template. Quantitative PCR (qPCR) was performed using Brilliant III Ultra-Fast SYBR Green QPCR Master Mix (Agilent Technologies) in a Stratagene Mx3000P RT-PCR instrument. Reactions were set up as mentioned earlier, in triplicates. Cycle threshold (Ct) values were recorded for all samples. Additionally, diluted samples were prepared using ${1}\,{\upmu \hbox {l}}$ of 1:100 dilution of cDNA in filtered lake water as template for ${20}\,{\upmu \hbox {l}}$ PCRs for 35 cycles. These samples are denoted ‘1:100’.

### Electrochemical biosensor

The PCB electrodes were manufactured using commercially available, low-cost electroless nickel immersion gold (ENIG) process without additional gold electroplating. The ENIG PCB electrode specifications are detailed in our previous work^11^. Conventional cleaning recipes for electrodes, such as using piranha solution or cyclic voltammetry with sulphuric acid are not recommended for ENIG PCB electrodes, as they may cause stripping of the thin gold layer (thickness $\approx$ $100\,\hbox {nm}$) and expose the underlying copper layer that is susceptible to corrosion^21–25^. The electrodes were therefore cleaned using lint-free wipes dampened with IPA. The samples to be measured are incubated with ${50}\,{\upmu \hbox {M}}$ MB at ${4}\,{^{\circ }\hbox {C}}$ for ${1}\,{\hbox {h}}$ to facilitate intercalation. In our previous work, we observed that the sensitivity and linearity of the sensor improved by increasing the MB concentration^11^. Based on the optimisation reported in our earlier work, we used ${50}\,{\upmu \hbox {M}}$ MB concentration for intercalation with DNA in this study. Electrochemical detection of double stranded DNA (ds-DNA) can be achieved using anionic or cationic intercalators. Although anionic intercalators detect DNA with better selectivity they require overnight incubation, resulting in longer assay time. Cationic intercalators such as MB, on the other hand, require lesser incubation time of approximately ${1}\,{\hbox {h}}$ for electrochemical detection of ds-DNA^6^. Each measurement involved dispensing ${5}\,{{\upmu \hbox {l}}}$ of the sample to be tested on the electrode, followed by cleaning with IPA dampened wipes before performing the next measurement with another sample. Each sample was tested on 5 different electrodes, unless otherwise mentioned. DPV and CV measurements were performed using PalmSens Sensit Smart potentiostat, and PSTrace software was used for potentiostat configuration and data acquisition, including peak current calculation. The following settings were used for DPV and CV measurements:

- DPV: equilibration time = $8\,\hbox {s}$, voltage step = $3\,\hbox {mV}$, pulse voltage = $25\,\hbox {mV}$, pulse duration = $50\,\hbox {ms}$, scan rate = ${20}\,\hbox {mV/s}$
- CV: equilibration time = $8\,\hbox {s}$, voltage step = $3\,\hbox {mV}$, scan rate = ${300}\,\hbox {mV/s}$

## Results and discussion

### DNA fragments of dissimilar lengths exhibit different mechanisms in voltammetric response

**Figure 2:**
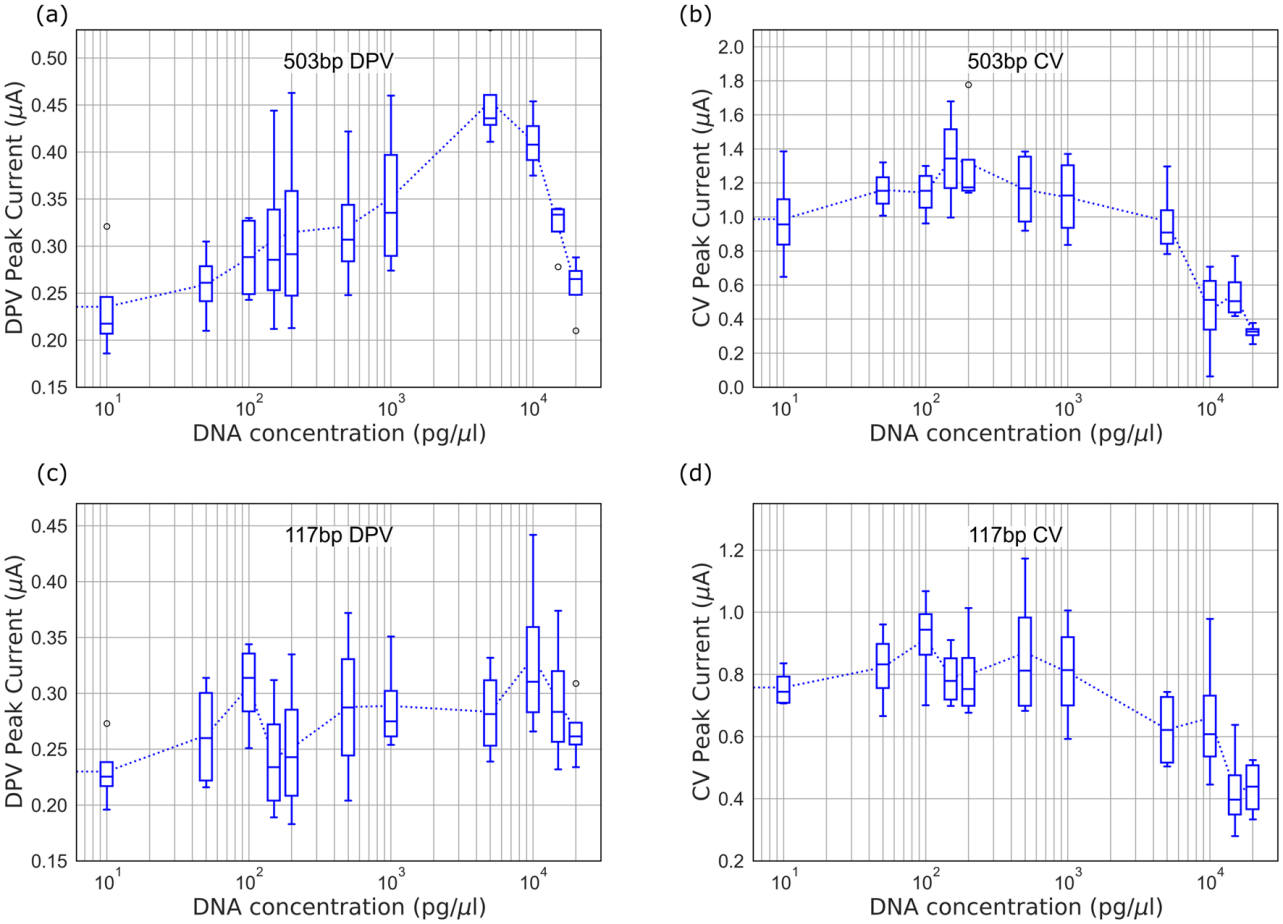
Peak current obtained from voltammograms for DNA complexed with ${50}\,{\upmu \hbox {M}}$ MB: (**a**) $503\,\hbox {bp}$ DPV, (**b**) $503\,\hbox {bp}$ CV, (**c**) $117\,\hbox {bp}$ DPV, (**d**) $117\,\hbox {bp}$ CV.

DPV and CV voltammograms were obtained on ENIG PCB electrodes for ${50}\,{\upmu \hbox {M}}$ MB complexed with DNA (concentration varying in the range 10–${20}\,{\hbox {ng}/{\upmu \hbox {l}}}$ i.e. 0.13–${0.26}\,{\upmu \hbox {M}}$ for $117\,\hbox {bp}$ and 0.03–${0.06}\,{\upmu \hbox {M}}$ for $503\,\hbox {bp}$). Representative voltammograms are shown in Fig. S1 in supplementary information. Figure 2 shows the results (peak current) obtained for DPV and CV measurements with gel purified PCR products. DPV measurements show higher sensitivity (change in current with increase in DNA concentration) as compared to CV measurements, since the background capacitive current in CV measurement hides the Faradaic current^26^. The data for each box in the box plots contains measurements from 5 electrodes. The same set of electrodes were used for all measurements, to avoid measurement errors due to electrode-to-electrode variations. We observed increasing trend in the peak current for DPV and CV measurements for lower concentrations of DNA, with the increase more pronounced for the longer ($503\,\hbox {bp}$) DNA fragment as compared to the $117\,\hbox {bp}$ fragment. This is consistent with the trend expected for adsorption on the electrode, reported in our previous work^11^. Adsorption of the MB-DNA complex facilitates charge transfer at the electrode, thus contributing to rise in peak current. Other studies have shown the influence of oligonucleotide size and sequence on MB-DNA intercalation^27–30^. The Guanine-Cytosine (GC) content of both the amplicons ($117\,\hbox {bp}$ and $503\,\hbox {bp}$) is approximately 50 %, suggesting that the differences observed are due to the amplicon length. However, for higher DNA concentrations ($>{2}\,{\hbox {ng}/{\upmu \hbox {l}}}$ for $503\,\hbox {bp}$ and $>{10}\,{\hbox {ng}/{\upmu \hbox {l}}}$ for $117\,\hbox {bp}$), we observed a decrease in peak current for both amplicons, in DPV as well as CV measurements. This is because MB saturates and intercalates between the base pair of the DNA, causing steric inhibition of redox activity of reducible groups in MB^31,32^.

### Presence of salt in sample reduces electrochemical sensitivity at lower DNA concentrations

**Figure 3:**
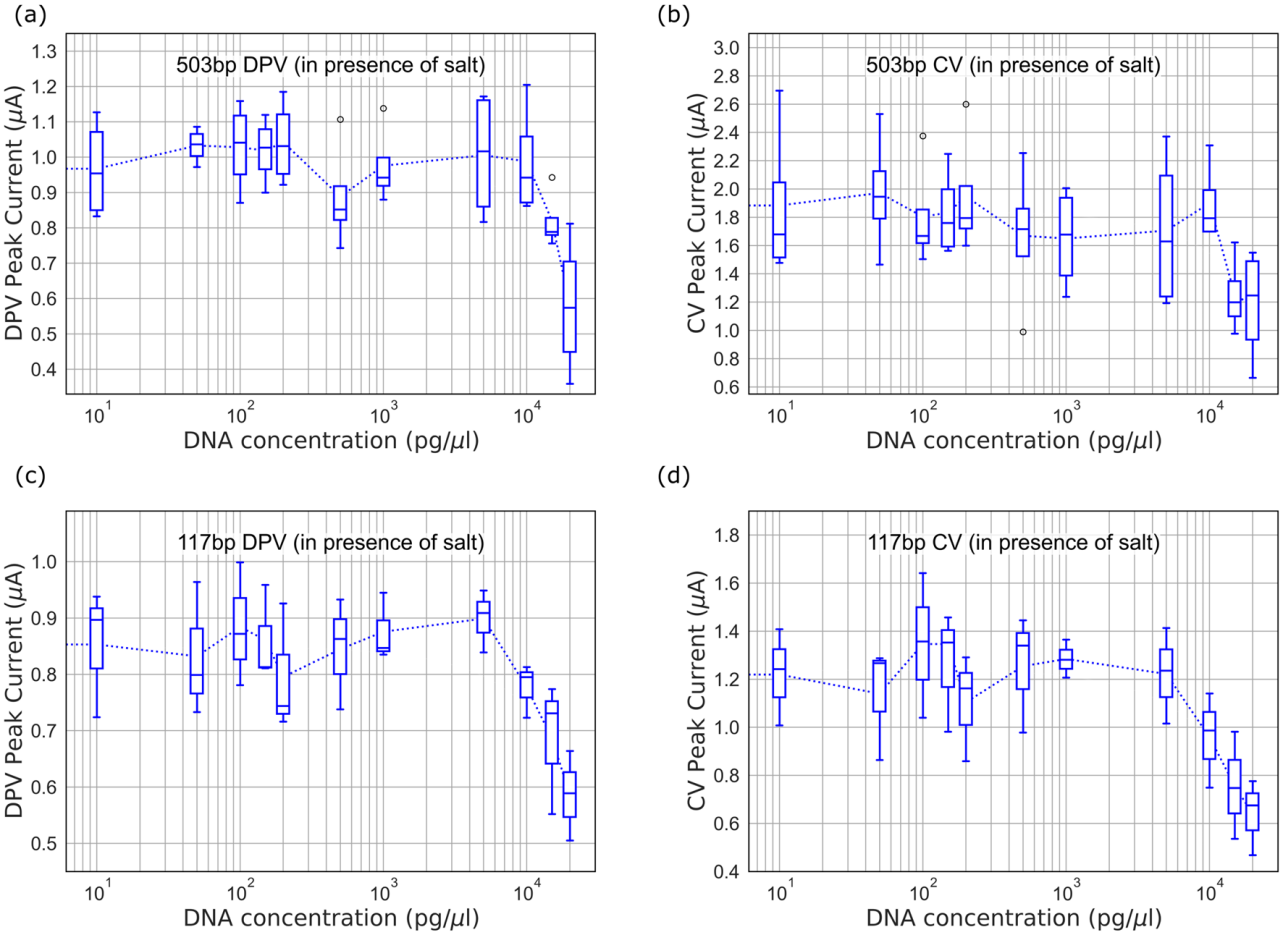
Peak current obtained from voltammograms for DNA complexed with ${50}\,{\upmu \hbox {M}}$ MB in presence of $2\,\hbox {mM}$ ${\hbox {MgCl}_2}$: (**a**) $503\,\hbox {bp}$ DPV, (**b**) $503\,\hbox {bp}$ CV, (**c**) $117\,\hbox {bp}$ DPV, (**d**) $117\,\hbox {bp}$ CV.

Salt present in PCR master mix can interfere with the electrostatic interactions between MB and DNA, and therefore the DPV and CV measurements on 5 electrodes were performed separately by adding $2\,\hbox {mM}$ $\hbox {MgCl}_2$ to the gel purified products with ${50}\,{\upmu \hbox {M}}$ MB, to study the impact of salt on the MB-DNA interaction. As seen in Fig. 3, we observe that the decreasing trend in peak current for higher DNA concentrations ($>{2}\,{\hbox {ng}/{\upmu \hbox {l}}}$ for $503\,\hbox {bp}$ and $>{10}\,{\hbox {ng}/{\upmu \hbox {l}}}$ for $117\,\hbox {bp}$), in both DPV and CV measurements, is not significantly impacted by addition of salt (see Fig. S2 in supplementary information for representative voltammograms). However, at lower DNA concentration, the sensitivity is greatly reduced by addition of salt, resulting in no significant change in current with DNA concentration. Other researchers have previously reported similar negative impact of salt on MB-DNA interactions and intercalation^33,34^. The $\hbox {Mg}^{2+}$ cation binds with the negative phosphate backbone of DNA thereby hindering the electrostatic interactions between MB and DNA^35^. At higher DNA concentrations, steric inhibition of redox active MB causes reduction in peak current, and therefore electrostatic interactions do not significantly influence the sensor response. The key takeaway is that such biosensors are more suitable for detection of higher DNA concentrations (few ${\hbox {ng}/{\upmu \hbox {l}}}$ or higher) for use-cases such as fully-automated processing of environmental water samples, where gel purification of the PCR products may not be feasible.

### Longer DNA results in more pronounced change in optical absorption for higher concentrations

**Figure 4:**
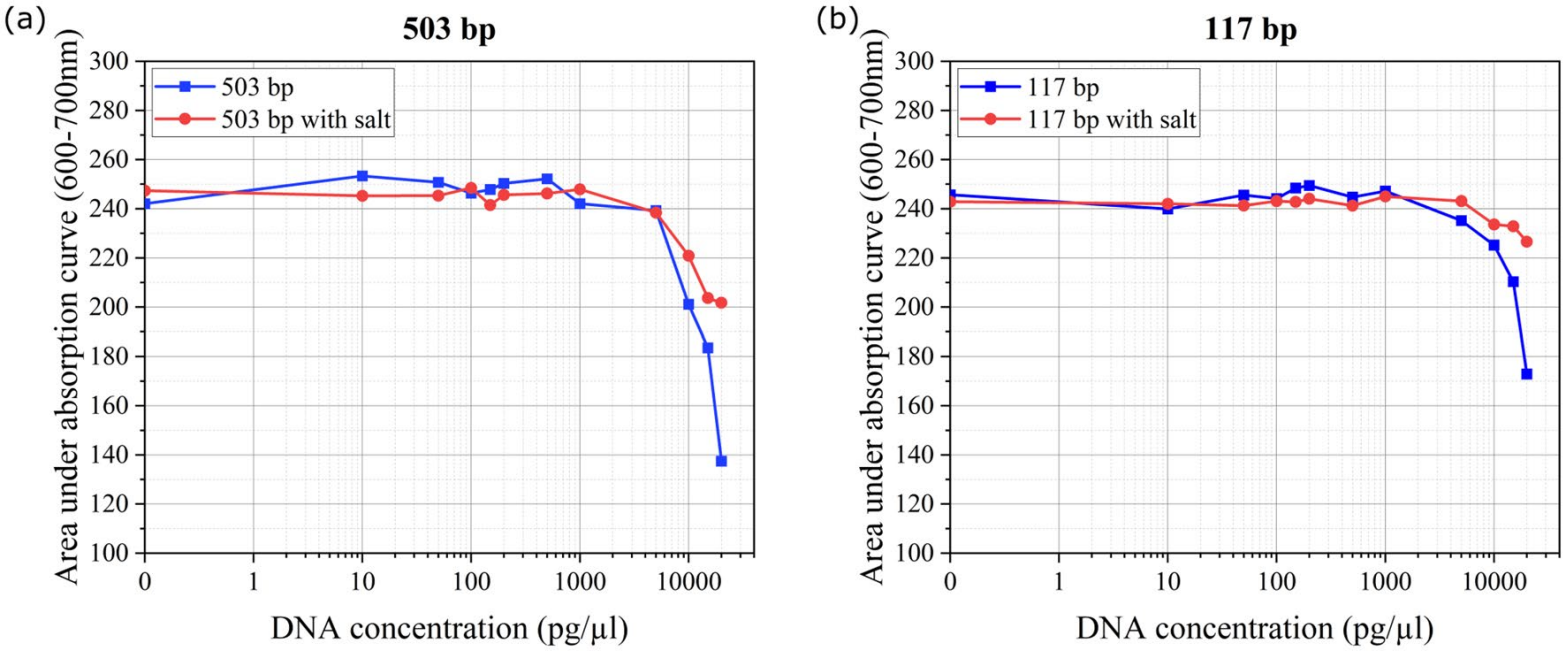
Area under the absorption curve for wavelength range 600–700 $\hbox {nm}$ for various concentrations of DNA complexed with ${50}\,{\upmu \hbox {M}}$ MB: (**a**) $503\,\hbox {bp}$ with and without added salt ($2\,\hbox {mM}$ $\hbox {MgCl}_2$), (**b**) $117\,\hbox {bp}$ with and without added salt ($2\,\hbox {mM}$ $\hbox {MgCl}_2$). ${0}\,{\hbox {pg}/{\upmu \hbox {l}}}$ DNA concentration corresponds to ${50}\,{\upmu \hbox {M}}$ MB sample without DNA.

To further validate the results discussed above, we performed optical measurements using a UV/Vis spectrophotometer (Thermo Scientific Multiskan GO), with ${50}\,{{\upmu \hbox {l}}}$ of sample used for each measurement. The absorption signature decreases with increase in DNA concentration, as seen in the trend for area under the absorption curve for wavelength range $600\,\hbox {nm}$ to $700\,\hbox {nm}$, shown in Fig. 4 (absorption spectra shown in Fig. S3 in supplementary information). There is no noticeable difference in the absorption for samples containing DNA compared to sample containing only MB, for DNA concentration lesser than ${1}\,{\hbox {ng}/{\upmu \hbox {l}}}$ (for both $503\,\hbox {bp}$ and $117\,\hbox {bp}$ length fragments), indicating the absence of steric inhibition of redox active MB. At higher DNA concentrations, we observed gradual decrease in the absorption signal, and noticed that the extent of reduction in absorption is lesser in presence of salt. These results are attributed to the molecular interactions with base stacks in the DNA hybrid, and steric inhibition. Our results are in concurrence with reports on spectroscopic studies of MB-DNA intercalation in literature, that have associated the hypochromism with decrease in the energy level of $\pi$–$\pi ^*$ electron transition due to intercalation^36–38^.

### Longer DNA fragment from Phi6 bacteriophage isolated from lake water can be detected with high sensitivity using unmodified PCB electrode

**Figure 5:**
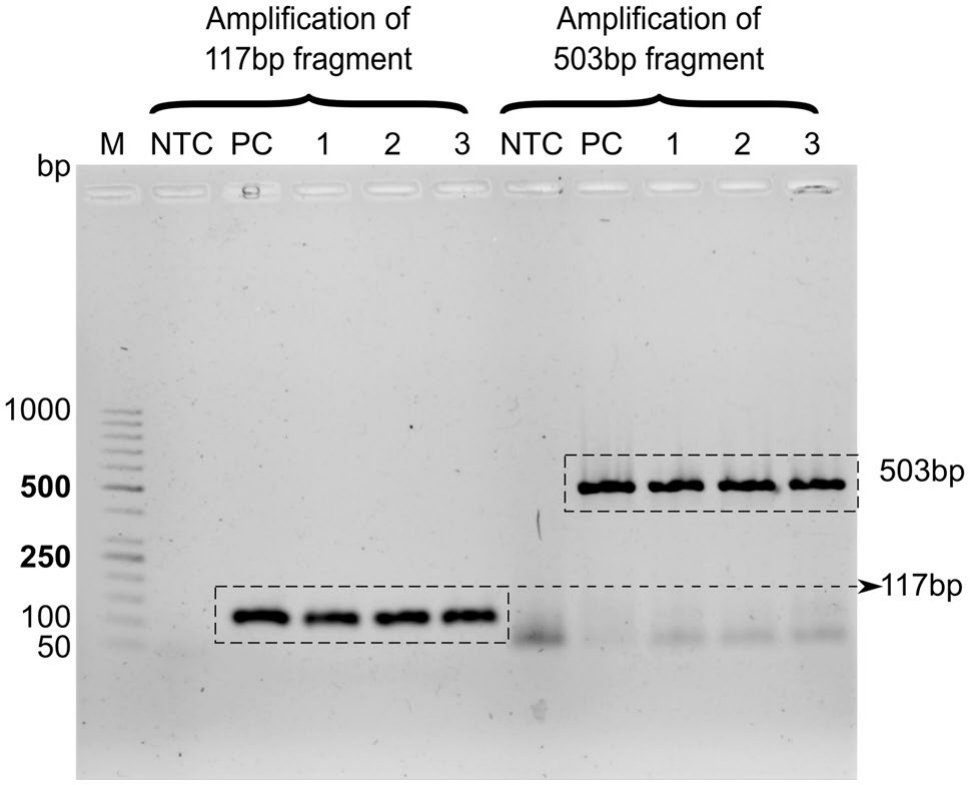
Agarose gel electrophoresis for bacteriophage Phi6: $117\,\hbox {bp}$ and $503\,\hbox {bp}$ length PCR products from lake water sample. M-DNA marker; NTC-no-template control containing the primers for respective amplicons; PC-positive control; 1, 2, 3- undiluted (1:1) spiked lake water samples in triplicate. A band is seen at $\approx 50\,\hbox {bp}$ due to unused oligos in the $503\,\hbox {bp}$ lanes.

We evaluated the utility of our sensor with water sample from Powai Lake spiked with Phi6 bacteriophage. The concentration of the RNA isolated from the phage spiked water samples ranged from 15.8–${19.4}\,{\upmu \hbox {g}/\hbox {ml}}$, while the RNA isolated from purified phage suspension was estimated to be ${1945}\,{\upmu \hbox {g}/\hbox {ml}}$, giving a recovery efficiency of approximately 1 %. RNA was reverse transcribed to cDNA and used as template for both PCR and qPCR. The products sizes were confirmed by agarose gel electrophoresis (Fig. 5) before testing with the sensor. These samples were not gel purified, and hence contain all components of PCR, along with target amplicons. Ct values recorded during qPCR (Table 1) showed a correlation with concentration of RNA isolated from the corresponding spiked water sample. Ct values reveal the number of cycles required for the fluorescence signal to exceed the threshold or background signal. Higher Ct value denotes lower template concentration, and vice-versa. The Ct values obtained for NTC samples were very high as expected. A difference of $\approx 3$ Ct value between the positive control and test samples further indicates each test sample has approximately 1 % of template compared to that of positive control. We have previously discussed that longer amplicons result in better sensitivity. The amplification of longer fragments isolated from heterogeneous environmental samples is challenging given the shortcomings due to low efficiency of virus concentration, and RNA degradation. However, with our virus concentration and PCR amplification protocols, we were able to successfully amplify the $503\,\hbox {bp}$ fragment for electrochemical sensing.

**Table 1:** Ct values of samples as measured by qPCR

	Ct value for $117\,\hbox {bp}$	Ct value for $503\,\hbox {bp}$
No template control (NTC)$^{1}$	$31 \pm 0.04$	$30.75 \pm 0.44$
Positive control (PC)$^{2}$	$17.87 \pm 0.17$	$16.73 \pm 0.12$
Test sample-1	$20.65 \pm 0.11$	$19.41 \pm 0.08$
Test sample-2	$20.37 \pm 0.07$	$19.35 \pm 0.07$
Test sample-3	$21.1 \pm 0.28$	$20.51 \pm 0.2$

$^1$ NTC has no template added in the reaction.

$^2$ Positive control has cDNA isolated from purified Phi6 bacteriophage particles as template.

**Figure 6:**
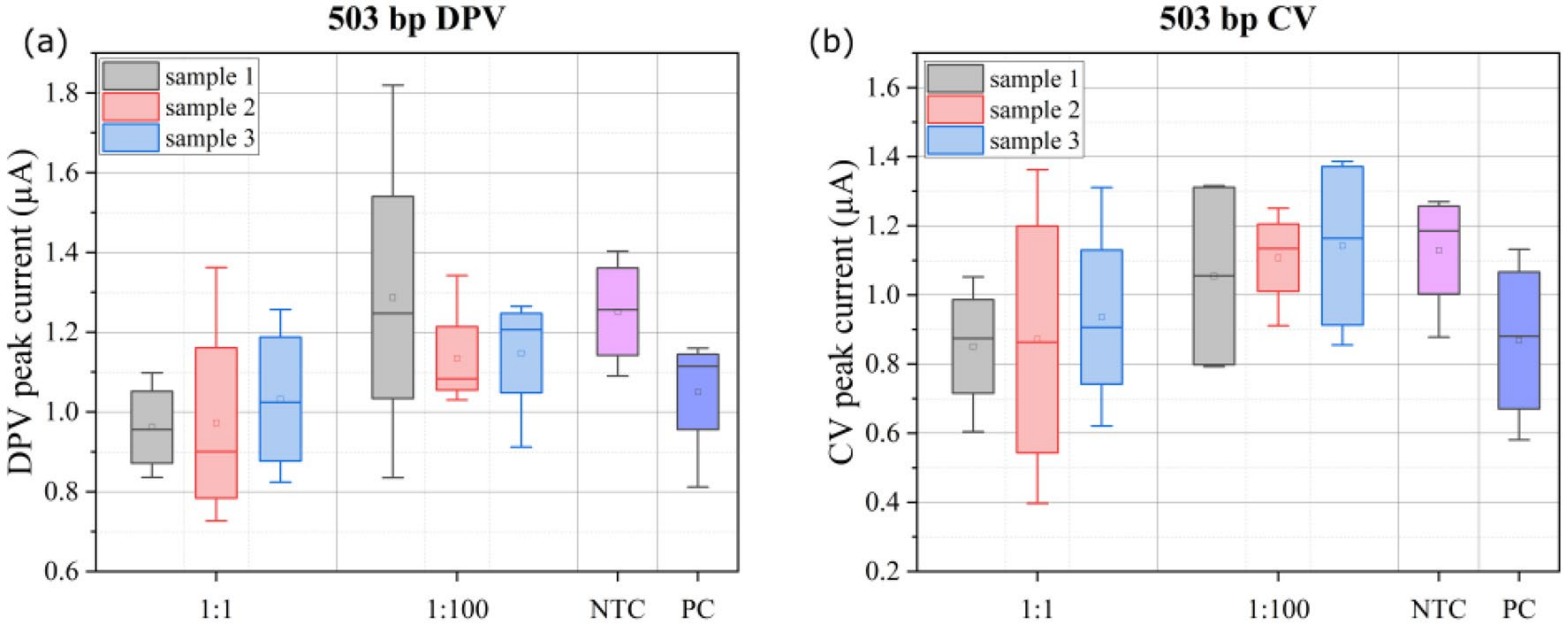
(**a**) DPV, and (**b**) CV peak currents for electrochemical detection of $503\,\hbox {bp}$ fragment from lake water sample. Measurements are performed in triplicates for the test samples, and compared to no-template control (NTC) and positive control (PC).

Figure 6 show the electrochemical sensor results obtained for $503\,\hbox {bp}$ fragment amplicons, both with undiluted cDNA as template (1:1) and hundred-fold diluted cDNA as template (1:100) for PCR, compared with NTC and PC (see Fig. S4 in supplementary information for representative voltammograms). Each box in the box plots in Fig. 6 comprises of measurements of triplicate samples on 5 electrodes. The same electrodes were used for measuring all samples, to avoid errors due to electrode-to-electrode variation. DPV measurements show better resolution for distinguishing test and PC samples from NTC, as compared to CV measurements, due to hiding of the Faradaic current by background capacitive current in the latter, as discussed before. For the longer amplicon we observed that the negative control (NTC) resulted in higher CV and DPV peak current relative to the positive control, whereas the positive and undiluted test samples revealed similar peak heights for DPV peak current. The mean and median value of the measurements for each undiluted (1:1) test sample and PC are clearly resolved from the sensor output for NTC samples, while the resolution for samples with 1:100 dilution are not as highly pronounced. For hundred-fold dilution of the cDNA, we did not observe any band during gel electrophoresis (lane not shown in Fig. 5) and the corresponding DPV and CV peak currents were similar to NTC, as expected. The results for $117\,\hbox {bp}$ fragment are presented in supplementary information. The negative control prompts an electrochemical response from the PCB sensor due to free MB adsorbing on the electrode, and the interaction of MB with single stranded primer oligonucleotides. Thus, each time a sample is to be tested, one must run a negative control and compare the peak current for test sample to that obtained for the negative control in order to achieve a differential (relative) measurement^39,40^ to classify the test sample as positive or negative.

### Discussion

Our findings illustrate different mechanisms that influence electrochemical sensor performance for different length of amplicons at varying DNA, concentrations validated by optical measurements using UV/Vis spectrophotometer. Our observations highlight the insight that longer DNA fragments up to $\approx$ $500\,\hbox {bp}$ can be detected with higher sensitivity, and the presence of salt in the sample does not compromise sensitivity for higher DNA concentrations (few ${\hbox {ng}/{\upmu \hbox {l}}}$ and beyond. Additionally, we studied the impact of different types of samples including gel purified amplicons with and without added salt, and spiked lake water samples, on DPV and CV measurements. We observed that DPV offers better resolution as compared to CV, since the background capacitive current also impacts CV measurement, rendering it less sensitive.

Amplification of longer fragments depends on integrity of the viral genomic RNA. Several studies have shown that amplification of longer fragments is not always efficient due to degradation of RNA in the environment and possibility of shearing during isolation^11,41–44^. We observed that PEG-based virus concentration method works better than aluminium hydroxide based virus concentration method to concentrate bacteriophage Phi-6 spiked in lake water samples. Demonstration of ability to detect long DNA fragment overcomes the requirement of multiplex PCR for amplifying multiple shorter length templates, and reduces the possibility of cross-specificity.

Biological samples are scarce, and therefore designing a biosensor that requires minimal sample for testing is desirable. The ENIG PCB electrodes used in this study require only $${5}\,{{\upmu \hbox {l}}}$$ of sample for testing to cover the active area of the electrode. Moreover, the same electrode can be reused after cleaning, before dispensing the next sample. The amplified sample does not require addition of any chemicals other than methylene blue, an inexpensive and commonly available chemical. Since the cost of manufacturing of each electrode is approximately USD$0.55 (i.e. INR ₹ 40), this biosensor can be a cost effective alternative to existing detection techniques. Table 2 shows a comparison of this work with other sensors for long DNA fragments in heterogeneous samples reported in literature.

The main limitation of this method is the possibility of non-specific amplification in heterogeneous samples such as wastewater and lake water, or with low purity primers, given the reliance of the MB based electrochemical detection scheme on PCR for specificity. To improve the sensitivity of the electrochemical detection method using unmodified ENIG PCB electrodes for DNA detection with unpurified PCR products, it is necessary to better understand errors introduced by the ununsed dNTPs and primers, optimise the reaction conditions and testing protocols. Measurement of auxiliary physicochemical parameters such as pH, temperature and biological oxygen demand (BOD) of the water samples may also be necessary to improve the accuracy of measurements.

**Table 2:** Comparison of this work with other biosensors for sensing long DNA fragments from heterogeneous samples

Reference	Sensing mechanism	Sensor/substrate	Test sample	Amplicon length
Ramirez et al.^10^	LAMP based electrochemical	Screen printed electrode	SARS-CoV-2 S and N gene in wastewater samples	$203\,\hbox {bp}$, $165\,\hbox {bp}$
Kumar et al.^11^	Electrochemical	ENIG finish PCB with no surface modification	Total RNA sample from wastewater spiked with SARS-CoV-2 RNA	$72\,\hbox {bp}^{1}$
Rosa et al.^45^	Nested RT-PCR	NA	SARS-CoV-2 spike protein in clinical and sewage samples	$300\,\hbox {bp}$
Ali et al.^46^	RT-LAMP$^{2}$ coupled CRISPR-Cas12	NA	SARS-CoV-2 RNA spiked sample	$200\,\hbox {bp}$
Baccari et al.^47^	TaqMan qPCR assay	NA	*S. negevensis* (ATCC VR-1471/Z) in water from wastewater treatment ponds and swimming pools	$116\,\hbox {bp}$
Yang et al.^48^	Electrochemical	Ferrocenyl (Fc) dsDNA intercalator (redox marker) and ssDNA/MCH immobilised gold electrodes	Human-specific mitochondrial DNA (mtDNA) from raw untreated wastewater	$195\,\hbox {bp}$
Yang et al.^49^	LAMP assay$^{2}$	Lateral flow device	Human-specific mtDNA from raw untreated wastewater	$\approx 200\,\hbox {bp}$
Kaarj et al.^50^	LAMP assay$^{2}$	Paper microfluidic device	Zika virus in tap water samples	$230\,\hbox {bp}^{3}$
Chandra et al.^51^	RT-qPCR	NA	Dengue, yellow fever, Zika and murine hepatitis viruses in untreated wastewater	$< 110\,\hbox {bp}$
This work	Electrochemical	ENIG finish PCB with no surface modification	Lake water spiked with bacteriophage Phi6	$503\,\hbox {bp}$

$^1$ $943\,\hbox {bp}$ fragment obtained from PCR amplification using control plasmid as template was detected, but could not be efficiently isolated and amplified from wastewater sample.

$^2$ Loop-mediated isothermal amplification

$^3$ Primer sets for NS5 gene sequence in ZIKV adapted from Tian et al.^52^

## Conclusion

In summary, we have presented a low-cost electrochemical ENIG PCB sensor for detection of viruses from environmental (lake water) samples. Unlike electrodes with immobilised oligonucleotides that need low-temperature storage to retain sensitivity, or custom fabricated substrates for DNA sensing^53,54^, our technology utilises unmodified PCB electrodes, with longer shelf life and no specific storage needs, and therefore suitable for developing automated sample processing and measurement solutions for deployment in LMICs. The biosensor utilises an inexpensive DNA intercalating redox dye (MB) for rapid detection of target amplicon. Since MB non-specifically binds to single and double stranded oligonucleotides, the non-specific amplification commonly found in environmental samples would reduce the specificity of such sensing method. Thus, the specificity of this test depends on the optimisation of primers and PCR reaction conditions. Further, the CV and DPV peak currents obtained from sample under test should be interpreted relative to response obtained from negative control (NTC) for every test. The electrochemical sensor design and approach presented in this work can be integrated with an auto-sampler for developing a fully automated and low-cost solution, that can collect and analyse samples, and communicate the findings back to the lab wirelessly.

## Supplementary Information

Supplementary Information.
