# Supplementary material for: Longer amplicons provide better sensitivity for electrochemical sensing of viral nucleic acid in water samples using PCB electrodes
Source: Sci Rep. 2022 May 25;12:8814. doi: 10.1038/s41598-022-12818-w (PMC9130999; doi:10.1038/s41598-022-12818-w)
Supplement: Supplementary file 1 — Supplementary Information. [file 41598_2022_12818_MOESM1_ESM.pdf]

Supplementary Information

Longer amplicons provide better sensitivity for electrochemical sensing of viral nucleic acid in water samples using unmodified PCB electrodes

Shruti Ahuja,[a] M. Santhosh Kumar,[b] Ruchira Nandeshwar,[c] Kiran Kondabagil[b*] and Siddharth Tallur[c*]

[a]Centre for Research in Nanotechnology & Science (CRNTS), IIT Bombay, Mumbai 400076, India.
[b]Department of Biosciences and Bioengineering, IIT Bombay, Mumbai 400076, India.
[c]Department of Electrical Engineering, IIT Bombay, Mumbai 400076, India.

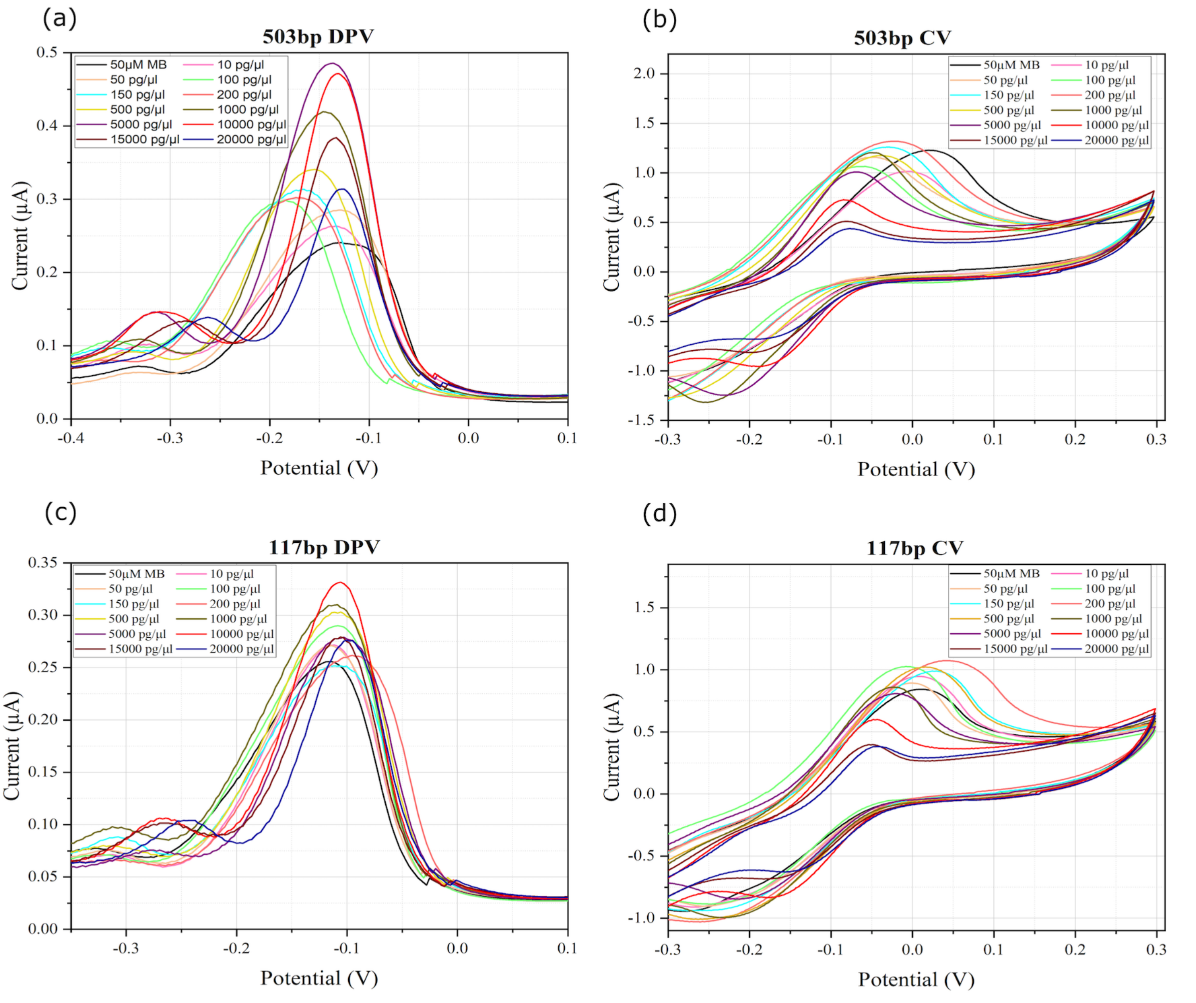

Figure. S1: Representative voltammograms obtained on 1 electrode for DNA complexed with 50 µM MB: (a) 503 bp DPV, (b) 503 bp CV, (c) 117 bp DPV, (d) 117 bp CV.

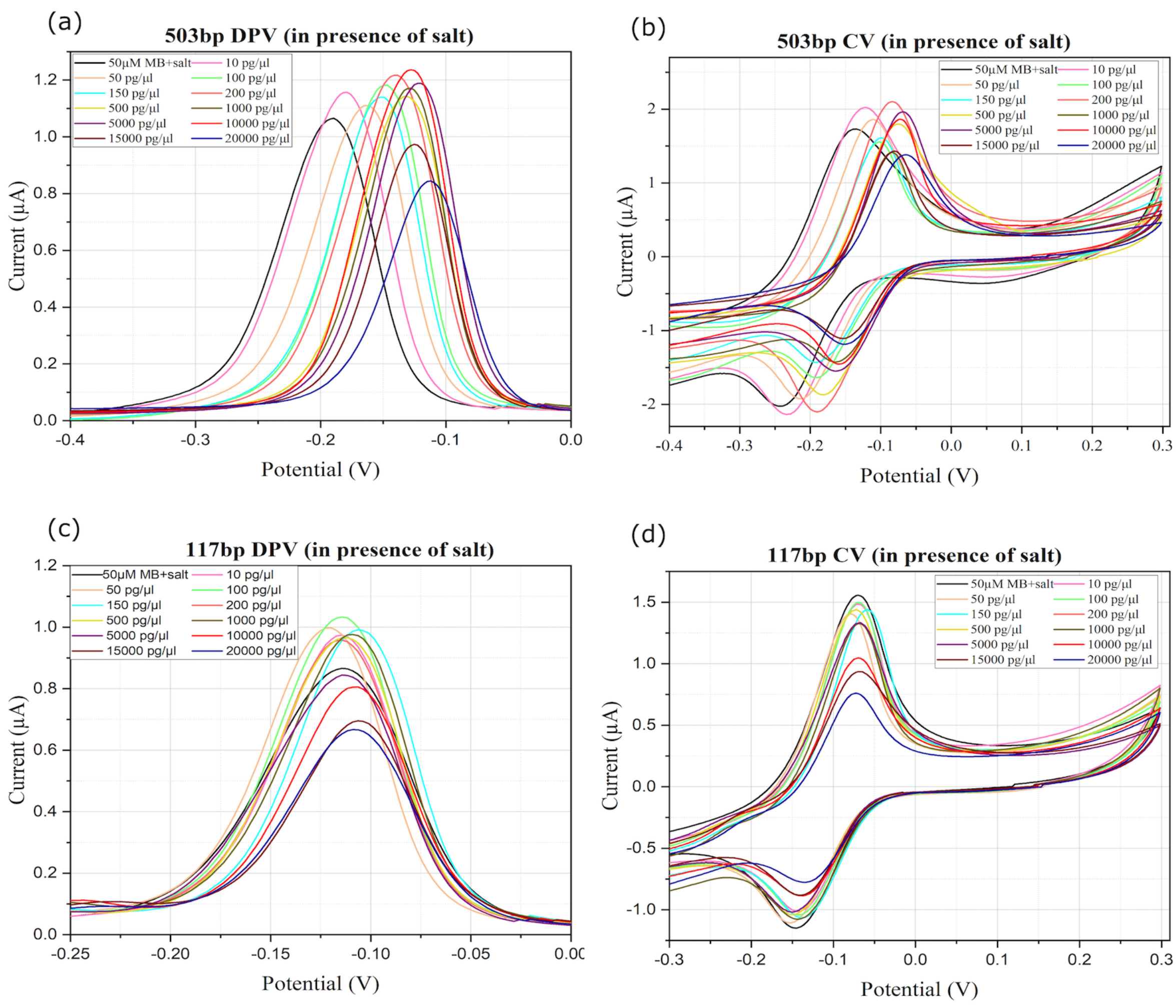

Figure. S2: Representative voltammograms obtained on 1 electrode for DNA complexed with 50 µM MB in presence of 2 mM $MgCl_2$: (a) 503 bp DPV, (b) 503 bp CV, (c) 117 bp DPV, (d) 117 bp CV.

Longer DNA exhibits pronounced change in optical absorption for higher concentrations

We performed optical measurements for MB-DNA samples for both 117 bp and 503 bp fragments (with and without added salt), using a UV/Vis spectrophotometer (Thermo Scientific Multiskan GO). The changes in absorption spectra of MB complexed with DNA are shown in Figure S3. The absorption signature decreases with increase in DNA concentration. There is no noticeable difference in the spectra for samples containing DNA compared to sample containing only MB, for DNA concentration lesser than 1 ng/µL (for both 503 bp and 117 bp length fragments), indicating the absence of steric inhibition of redox active MB. At higher DNA concentrations, we observed red shift and gradual decrease in the absorption peak intensity at ≈668 nm. The decrease in peak intensities is more profound in the longer fragment revealing the dependence of length on oligonucleotide length on MB-DNA interactions. Our results are in concurrence with reports on spectroscopic studies of MB-DNA intercalation in literature, that have associated the hypochromism with decrease in the distance between intercalated MB and DNA base pairs that consequently decreases the energy level of $\pi - \pi^*$ electron transition [1–3].

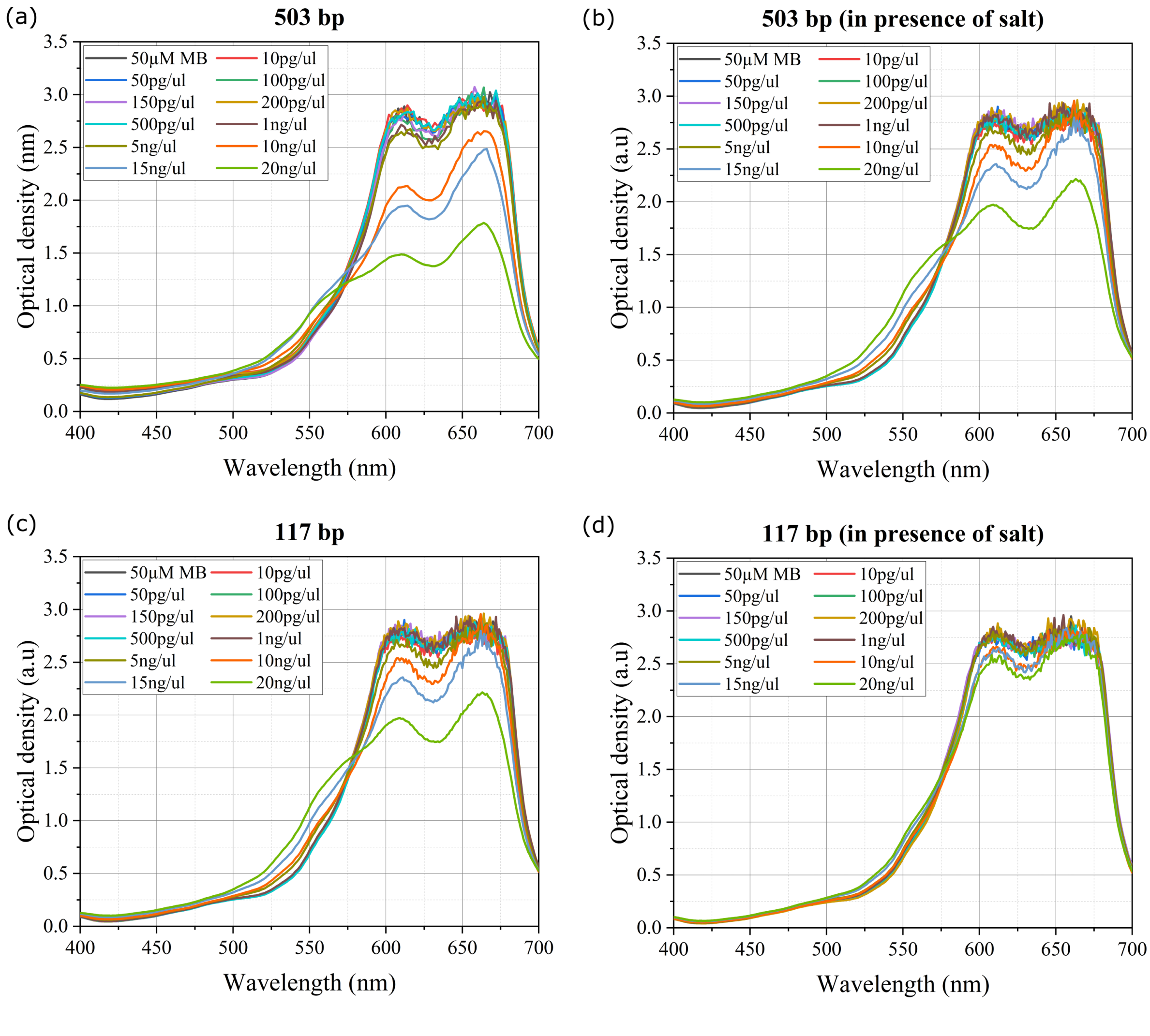

Figure. S3: Absorption spectra obtained from UV/Vis spectrophotometry for various concentrations of DNA complexed with 50 µM MB: (a) 503 bp with no added salt, (b) 503 bp in presence of 2 mM $MgCl_2$, (c) 117 bp with no added salt, (d) 117 bp in presence of 2 mM $MgCl_2$. In each panel, the '50 µM MB' curve represents sample without DNA.

Electrochemical detection of 117bp fragment from phage Phi6 concentrated from lake water

For the shorter 117 bp fragment, we observed that the DPV and CV peak currents for positive control (PC) and undiluted lake water samples (1:1) were higher than the negative control (Figure S4 and Figure S5), as opposed to the results observed for the longer 503 bp fragment reported in the manuscript. These results appear to follow the trend observed for peak current in experiments performed with gel purified amplicons at lower concentration, without added salt. Magnesium chloride acts as a co-factor for Taq polymerase, and could possibly get used up in the amplification reaction along with other PCR master mix reagents, thus explaining the trend in the current. Although one cannot rule out errors due to residual oligos/primers from amplification reaction, the CV peak current output values for PC, undiluted (1:1), and hundred-fold diluted (1:100) samples are well-resolved from NTC, consistent with the trend we reported in previous work for 72 bp long N1 fragment from SARS-CoV-2 amplified from simulated wastewater spiked with viral RNA [4], and warrant further investigation.

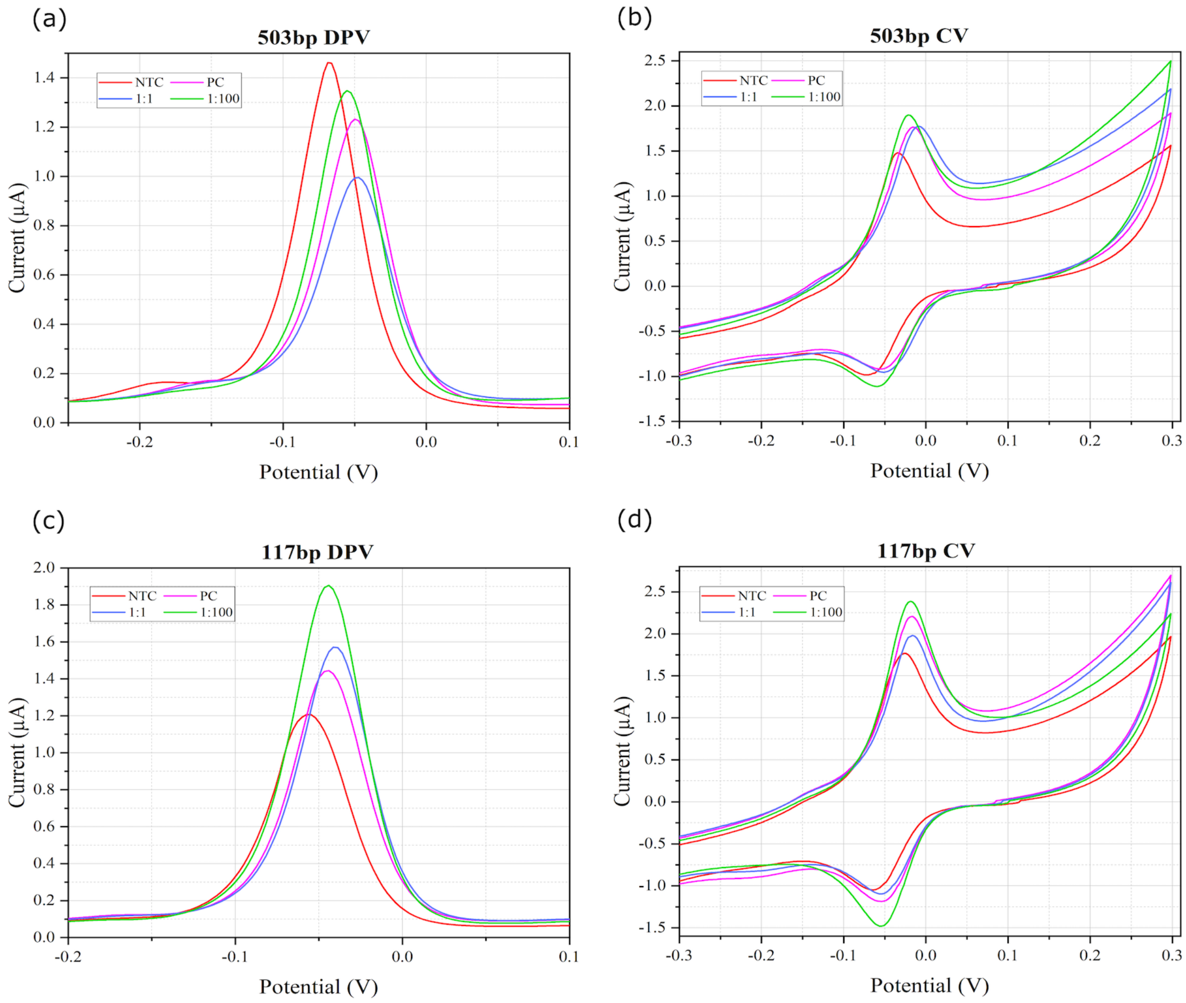

Figure. S4: Representative voltammograms obtained on 1 electrode for lake water samples complexed with 50 µM MB : (a) 503 bp DPV, (b) 503 bp CV, (c) 117 bp DPV, (d) 117 bp CV.

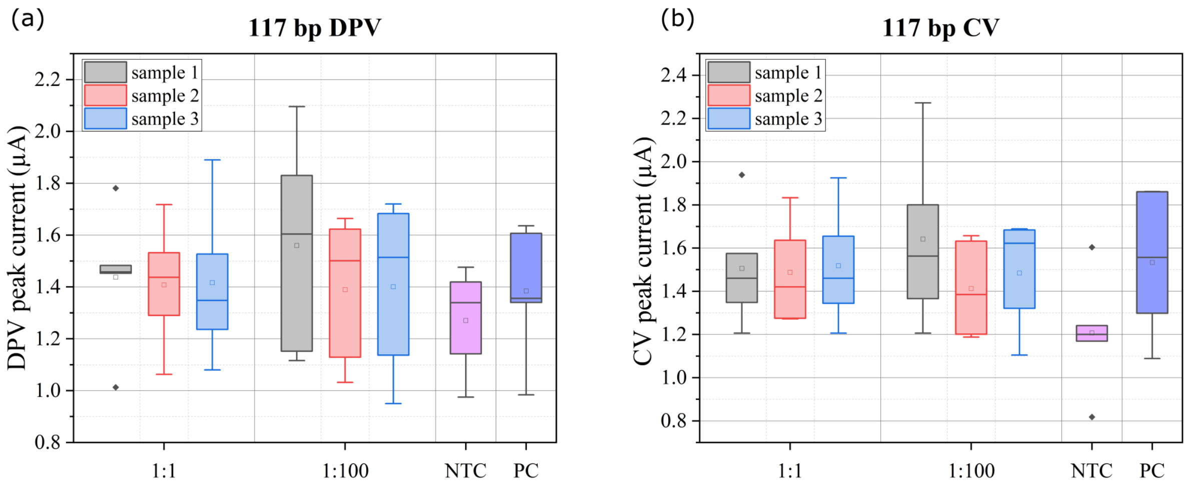

Figure. S5: (a) DPV, and (b) CV peak currents for electrochemical detection of 117 bp fragment from lake water sample. Measurements are performed in triplicates for the test samples, and compared to no-template control (NTC) and positive control (PC).

References

[1] MJ Simons. Interaction of methylene blue with DNA. A polarographic study. *Transactions of the Faraday Society*, 64:727–732, 1968.

[2] Reza Hajian, Nafiseh Shams, and Afsaneh Parvin. DNA-binding Studies of Daunorubicin in the Presence of Methylene Blue by Spectroscopy and Voltammetry Techniques. *Chinese Journal of Chemistry*, 27(6):1055–1060, 2009.

[3] PO Vardevanyan, AP Antonyan, MA Parsadanyan, MA Shahinyan, and LA Hambardzumyan. Mechanisms for binding between methylene blue and DNA. *Journal of Applied Spectroscopy*, 80(4):595–599, 2013.

[4] MS Kumar, Ruchira Nandeshwar, Shailesh B Lad, Kirti Megha, Maheshwar Mangat, Adrian Butterworth, Charles W Knapp, Mara Knapp, Paul A Hoskisson, Damion K Corrigan, Andrew C Ward, Kiran Kondabagil, and Siddharth Tallur. Electrochemical sensing of SARS-CoV-2 amplicons with PCB electrodes. *Sensors and Actuators B: Chemical*, page 130169, 2021.
